# Singing versus conversation for wellbeing in dementia: A cross-over pilot study

**DOI:** 10.1177/13872877261485601

**Published:** 2026-09-11

**Authors:** Dewi Boessen, Marijn Hendricksen, Thomas Johannsen, Esmée Verwijk, Jenny T. van der Steen, Fleur L. Bouwer

**Affiliations:** 1 Department of Psychology, Brain and Cognition, University of Amsterdam, Amsterdam, The Netherlands; 2 Zorggroep Amsterdam Oost, Locatie Flevohuis, Amsterdam, The Netherlands; 3 Genetic Choir, Amsterdam, The Netherlands; 4 Department of Medical Psychology, 522567 Amsterdam University Medical Center, Amsterdam, The Netherlands; 5 Department of Public Health and Primary Care, 4501 Leiden University Medical Center, Leiden, The Netherlands; 6 Department of Primary and Community Care, Radboudumc Alzheimer Center, 6034 Radboud university medical center, Nijmegen, The Netherlands; 7 Cicely Saunders Institute, 4616 King's College London, London, UK; 8 Cognitive Psychology Unit, Institute of Psychology & Leiden Institute for Brain and Cognition, Leiden University, Leiden, The Netherlands

**Keywords:** Alzheimer's disease, dementia, music, music-based intervention, research design, singing

## Abstract

**Background:**

Music-based therapeutic interventions are a promising addition to conventional treatments for people with dementia, positively affecting mood and wellbeing. However, previous studies on music-based interventions show variability in design and results, hampering clear understanding of their beneficial effects. Particularly, the specific benefits of music compared to similar non-musical activities remain unclear.

**Objective:**

Here, we piloted the feasibility of a randomized cross-over design to isolate music-specific positive effects of an intervention from general, nonspecific effects of participating in a stimulating group activity, assessing outcomes both in people with dementia and caregivers.

**Methods:**

15 nursing home residents diagnosed with dementia participated in both a music and a conversation activity, which only differed with respect to the presence of music. Before and after five weeks of intervention, we assessed behavioral symptoms and caregiver burden, using the Neuropsychiatric Inventory (NPI-NH), and wellbeing, using the Quality of Life in Late-Stage Dementia (QUALID) scale. During the activities we assessed engagement (Menorah Park Engagement Scale; MPES) and affect (Observed Emotion Rating Scale; OERS).

**Results:**

The singing activity reduced professional caregiver burden, while the conversation activity increased participant wellbeing. We found no difference between music and conversation in eliciting engagement and affect during the activities.

**Conclusions:**

Tentatively, both singing and conversation activities can positively affect people with dementia, with neither clearly outperforming the other. Given our sample size, these findings need to be confirmed in larger studies. We discuss the feasibility of the design and implications for future studies examining music-specific positive effects on people with dementia.

## Introduction

Dementia, a group of several neurodegenerative disorders characterized by decline across diverse cognitive domains,^1^ is a highly prevalent condition in older adults, with increasing personal and societal costs. To alleviate neuropsychiatric symptoms associated with dementia, pharmacological interventions are often ineffective,^2^ potentially contributing to increased professional caregiver burden and stress.^3^ Evidence is accumulating for nonpharmacological approaches in the treatment of neuropsychiatric symptoms.^4^ In this context, music-based therapeutic interventions may be especially promising.

Despite broad deficits in cognition, music processing is somewhat spared in different forms of dementia.^5^ Moreover, music is known to induce pleasure in healthy adults.^6^ Thus, music may be both accessible to people with dementia and is a known tool for mood regulation. Indeed, evidence suggests that music-based interventions may reduce neuropsychiatric symptoms and improve the quality of life of people with dementia.^7–11^ Moreover, music-based interventions may positively influence executive functions,^12^ emotions and social engagement in people with dementia,^12,13^ and may reduce behavioral disturbances and professional caregiver burden.^14–16^

While there is thus evidence for positive effects of music-based interventions on people with dementia, results are often inconsistent, and evidence has been regarded low-quality and low-certainty.^4,17,18^ A recent meta-analysis found no evidence for positive effects of music-based interventions when compared to other activities on emotional wellbeing, depression, or problematic behaviour.^10^ Moreover, the mechanisms by which music-based interventions may assert their effect and the critical ingredients needed for an effect to occur are unclear.^4^ It has been suggested that non-specific effects (e.g., interaction with an interventionist—Hawthorne effect) may be (partly) driving the putative positive influence of music-based interventions.^17,19^ One series of studies looked at the effect of a music activity on the behavioral and emotional status of people with dementia,^15,20^ comparing the music activity to a cooking activity. Whereas the music activity was initially deemed more effective for reducing behavioral symptoms, when the control activity was made equally attractive, the differences disappeared,^19^ suggestive of non-specific effects driving the positive influence of the interventions. Recently, however, music therapy was shown to yield music-specific effects on emotions and engagement in people with dementia.^13^ Here, music therapy outperformed a conversation activity which was equated for duration, type of activity, interventionists, and content in eliciting constructive engagement and positive emotions (e.g., pleasure) in people with dementia during intervention sessions. However, in this study, behavioral symptoms and wellbeing outside of the sessions were not assessed. Hence, results from studies examining music-specific effects of music-based interventions are inconclusive.

In the current work, we present a pilot study designed to isolate the music-specific effects of a music activity on quality of life in people with dementia and on caregiver burden. Within the broad variety of music-based interventions, *music therapy* is sometimes differentiated from *music activities*. Music therapy, as used in Reschke-Hernández et al. (2023),^13^ is considered a structured, clinical approach in which a trained music therapist uses music to concentrate on specific goals, while adjusting the structure of sessions to patient needs.^21^ Often, music therapy focuses on individuals or small groups.^7^ Music activities, as implemented by Samson et al. (2015) and in the current study,^19^ encompass a broader range of interventions, from passive music listening to group singing, and implementation is not limited to only qualified music therapists.^8,22,23^ As such, music activities may be a more scalable form of music-based interventions than music therapy.^7^ In some cases, musical activities were found to outperform musical therapy in improving quality of life in people with dementia.^7^ Here, we focus on a musical activity for residents at a long-term care facility, implemented by trained singers,^24^ which can also be led by volunteers after a brief introduction session, making it a potentially low-cost and scalable intervention.^24^ During the activity, the singers use improvisation to tailor their song and activities to the individual participants,^24^ as is often also done in music therapy in people with dementia.^25^ As such, while not performed by certified music therapists, the activity incorporates core ideas from group music therapy, including the interactive connection with the provider and the therapeutic goal.^10,24^

The current study employed a randomized cross-over design, comparing the effects of the singing activity to the effects of a control activity in which the same interventionists as in the singing activity led an improvisational conversation activity. The format of the conversation control activity was identical to the singing activity in that duration, time of day, and engagement with the participants was the same. In the conversation sessions, the singers conversed as they normally would with the participants.^10^ We assessed engagement^26^ and positive affect during the activities, as well as behavioral symptoms and quality of life^10^ before and after multiple weekly sessions (for research examining possible improvements in memory and cognition due to musical interventions, see^12,27–30^). Additionally, we assessed possible effects on caregiver burden, as neuropsychiatric symptoms are associated with caregiver distress.^31^ Based on previous research,^13^ we expected music activities to outperform conversation in eliciting engagement and positive affect, in improving quality of life,^32,33^ and in decreasing behavioral symptoms^16,18^ and professional caregiver burden.^34–37^ In addition to a preliminary analysis of these dependent variables, in this randomized crossover pilot study, we assessed the feasibility of the study design, to increase understanding of how the interventions might work, and to inform future trials.

## Methods

### Study design

In this study we assessed the effects of two different activities, a singing activity and a conversation activity, on wellbeing and professional caregiver burden. We used a crossover design, allowing for the assessment of within-subject effects, and allowing us to offer both activities to all participants, as done previously^10,13^ (Figure 1). The activities took place 50 min per week for 5 weeks, with a 4-week wash-out period in between, to avoid carryover effects, for a total of 10 sessions per group (5 singing, 5 conversation). The activities for the two groups were conducted on the same afternoon with a small break in between for the interventionists. The order of activities was counterbalanced to avoid potential effects of time of the day (i.e., one week, the singing group was in the early afternoon and the conversation group was later in the afternoon, with the order reversed the next week). The full design included a baseline measurement during a week of standard care (week 0), 5 weeks of either singing or conversation activity (week 1–5), with a measurement of behavioral symptoms, professional caregiver burden, and wellbeing in the last week (end of week 5), 4 weeks of standard care as a wash-out period (weeks 6–9), with a second baseline measurement in the last week (end of week 9), followed by another 5 weeks with activities (weeks 10–14), with another measurement of behavioral symptoms, professional caregiver burden, and wellbeing in the last week (end of week 14, see Figure 1). The two groups were randomly assigned to starting with the singing or conversation activity in the first period. Hence, randomization was done at the group (unit) level, given the living arrangements of the participants.

**Figure 1.**
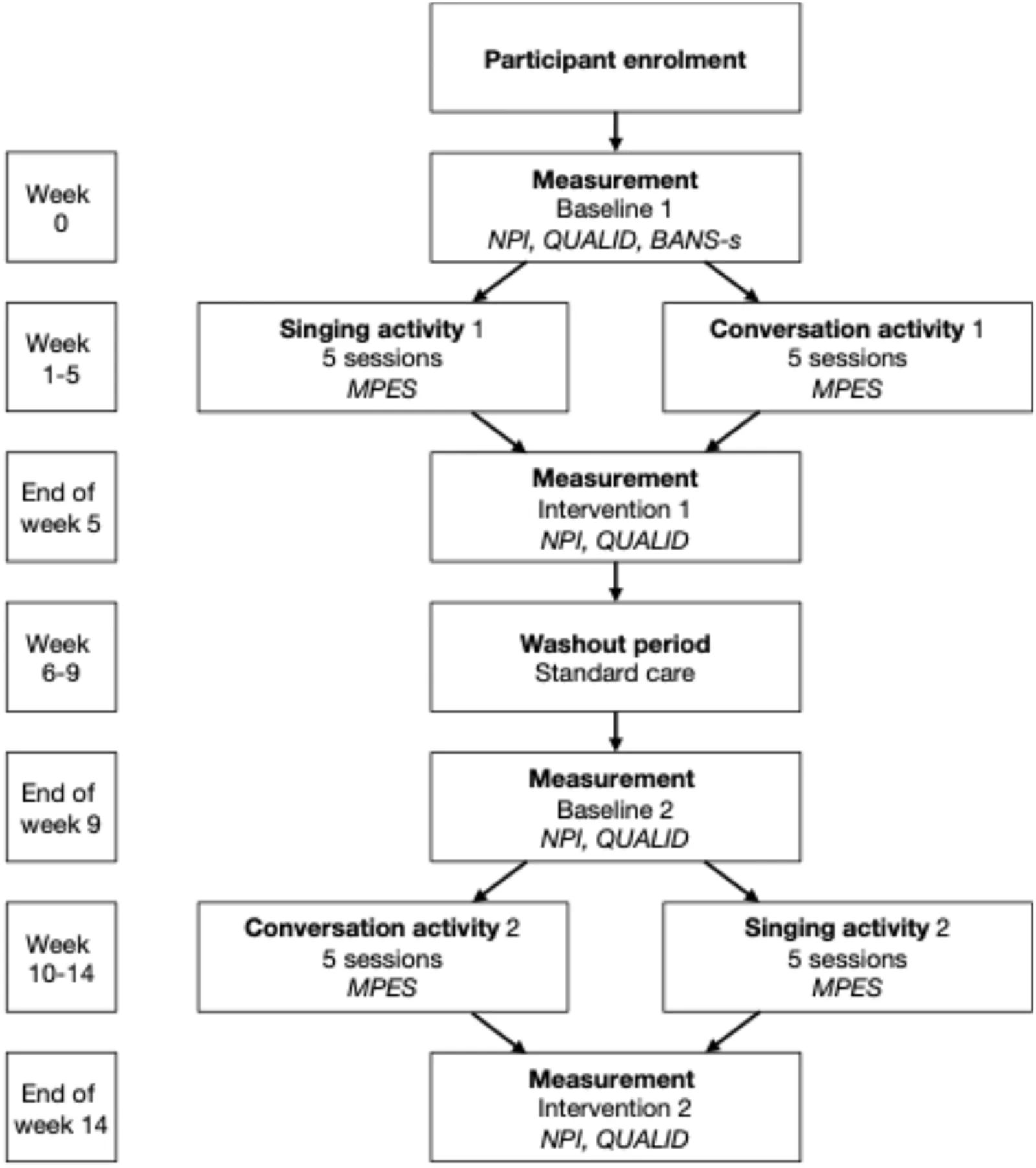
Participant flow diagram. We used a within-subjects crossover randomized design. NPI and QUALID were filled out about the week preceding the measurement (e.g., for Intervention 1, the questionnaires were filled out about week 5).

### Participants

Participants were recruited through a convenience sample, from 2 units in a nursing home psychogeriatric department in the Netherlands. Participants had not participated in the activities used in this study before (i.e., they were treatment-naïve). Where possible, participants were informed in simple terms about the project and gave their assent verbally. Additionally, family members of the residents were informed by email about the study and asked for written consent. When no family members were available, other legal representatives were approached for consent. As the study consisted of observation only of an activity that was already ongoing in other units in the nursing home, we were granted approval to enroll residents also when there was no response from family members and representatives. The protocol was approved by both the nursing home and the Ethics Review Board of the Faculty of Social and Behavioural Sciences of the University of Amsterdam, number FMG-3146.

Of 15 participants initially recruited, one died. The remaining 14 participants (5 males, and 9 females, 7 from each unit) were between 71 and 100 years old (mean age 83.6 years, SD = 9.0 years). Dementia types were Alzheimer's disease (n = 5), vascular dementia (n = 2), Parkinson's dementia (n = 1), frontotemporal dementia (FTD; n = 1), and unspecified dementia (n = 4). One participant had acquired brain injury, but given that the symptoms, demographics, and living circumstances of this participant were the same as those of the other participants, we included this participant.

### Dementia severity

The Bedford Alzheimer Nursing Severity-Scale (BANS-S) was used to assess severity of disease among the participants.^38^ The BANS-S was filled out once by nursing home personnel familiar with the participants at baseline (see Figure 1). The BANS-S comprises 7 items on a 4-point scale aimed at quantifying the degree of impairment associated with Alzheimer's disease. Scores on the BANS-S range from 7 to 28 points, with higher scores indicating greater disease severity. The BANS-S demonstrates robust validity and reliability for assessing the severity of Alzheimer's disease in nursing home residents.^39,40^ BANS-S scores of 17 or higher indicate severe dementia and roughly correspond to a Mini-Mental State Examination score of below 10. However, whereas the Mini-Mental State Examination shows floor effects in people with severe dementia, the BANS-S is discriminative, and hence, appropriate for our nursing home sample.^41^ Of the 14 participants, 6 participants scored 17 or higher on the BANS-S, indicating severe dementia, with the remainder of the participants scoring below 17, indicating mild to moderate dementia (overall range 7–20; M = 14.5; SD = 4.4). Only 1 participant obtained the minimum score of 7, and none the maximum score of 28, indicating that like in previous studies,^40^ the BANS-S did not show ceiling or floor effects in our nursing home sample. Participants in the two nursing home units also showed a similar range of BANS-S scores (unit 1: range 7–20, M = 14.4; unit 2: range 9–20, M = 14.4), indicating that both units housed a mixture of individuals differing in disease severity.^41^

### Dependent variables

Assessment of behavioral symptoms, professional caregiver burden, and wellbeing. Both before and after each activity period, behavioral symptoms and wellbeing were assessed (i.e., in total four times, see Figure 1) by nursing home personnel familiar with the participants. Nursing home personnel involved in assessing the behavioral symptoms and wellbeing were not informed of the nature of the two conditions. Initially, assessment was done through an interview by one of the researchers (MH) using the NPI-NH and QUALID. Once nursing home personnel was familiar with the questionnaires, where possible, they self-completed the assessments.

For behavioral symptoms and professional caregiver burden, the Neuropsychiatric Inventory Nursing Home Version (NPI-NH) was used,^37,42^ as was done previously in several studies evaluating the efficacy of music interventions in dementia.^10^ The NPI-NH assesses twelve behavioral and psychological symptoms commonly associated with dementia on frequency (0 till 4), severity (0 till 3), and associated caregiver distress (0 till 3). Here, we report the total score (i.e., a resident outcome), computed as the sum of frequency scores multiplied with severity scores for each symptom (range 0–144), as well as the caregiver distress (i.e., a nursing staff outcome, range 0–36). The NPI-NH has good internal consistency, inter-rater reliability, and construct validity.^37^ In the current study, we assessed symptoms present in the week preceding the time of the assessment.

The Quality of Life in Late-Stage Dementia (QUALID) scale was used to assess wellbeing as a single domain.^33^ The 11 QUALID items (5-point scale, total score range 11–55) include indicators of wellbeing and observable mood, such as sadness, fear, and irritability, as well as positive behaviors, such as smiling or engaging in enjoyable activities. Higher scores indicate a lower quality of life (wellbeing) for the individual with dementia. The QUALID has demonstrated strong inter-rater reliability, and has been validated through comparison with other established quality of life measures.^33^ The QUALID has been shown to validly index quality of life in people with dementia at different stages of the disease, from early to severe dementia.^43,44^ Moreover, the translation to Dutch has been validated through comparison with observed discomfort in people with severe dementia.^32^

Assessment of engagement with singing and conversation activities. During the activities (see Figure 1), the Menorah Park Engagement Scale (MPES) was used to assess engagement.^45–47^ The original scale consisted of four different, mutually exclusive, engagement items—constructive, passive, non-, and self-engagement.^46^ Here, we used the modified version of the MPES with the fourth item being “other” engagement, including self-engagement.^47^ In addition, as was done previously,^47^ we included items coding for observed affect from the Observed Emotion Rating Scale,^48^ as well as items coding for whether someone left the room or showed inappropriate behaviour.^47^ Finally, an item “song” was added to score the amount of singing by the singers during the activity. Hence, in total, we rated 11 items: constructive engagement, passive engagement, non-engagement, other engagement, inappropriate behavior, happiness, fear, anger, sadness, left the room, and song.

Data collection for the MPES and affect scales occurred in blocks of 5 min, with scores ranging from 0 (not observed) to 1 (observed less than half of the time) and 2 (observed more than half of the time), as was done previously.^47^ During the activities, participants were observed by two research assistants (DB and MH) seated in the corners of the room, who did not directly interact with the participants. Nine blocks of 5 min were coded by each observer during each 50-min intervention, starting a few minutes after the interventionists entered the room. All participants (7 per group) were thus observed at least once in each activity by each observer. The observers started with different participants, and observations were conducted with a rotation of participants every 5 min and over sessions (e.g., in session 1, observer 1 started with participant 1, and observer 2 started with participant 5; in session 2, observer 1 started with participant 2, and observer 2 started with participant 6), to obtain as much variability in the observed time respective to the start of a session as possible.

Assessment of feasibility for interventionists and treatment fidelity. We assessed the experience of the singers taking part in the study, who had to adjust the activity to conversation instead of singing, with a short questionnaire after every session, with a judgement on a 5-point Likert scale for 1) How much they sang during the session (ranging from “not at all” to “very much”), 2) How much contact they felt they had with the residents (“not at all” to “very much”), 3) How the residents responded to the session (“very negatively to very positively”), 4) Whether the residents were actively engaged with the session (“not at all” to “very much”), and 5) How satisfied they were with the session (“very unsatisfied” to “very satisfied”).

### Activities

Singing. The singing activity involved active music-making by the interventionists through using voice, sound, and improvisation, with participants joining in by their own accord. We used a method developed and carried out by a choir specialized in improvisational music, called the Genetic Choir. Especially for dementia care, they developed an intervention called Stem&Luister, in which improvisation is used as a design feature.^24^ Improvisation methods were specifically developed to be used in nursing home settings by the choir together with healthcare professionals, and have been implemented in nursing homes since 2020. The Stem&Luister intervention has already been studied extensively from a qualitative, and anthropological perspective by Gysels et al. (2024).^24^ Here, we focus on a quantitative and (neuro)psychological perspective.

In the sessions, four singers entered a room where participants were already present. Singers engaged with the participants, using diverse types of improvised song, activities centered around song, and music making from the “found sounds” that were already audible in the room. For example, the singers might start a song with a simple melody and encourage patients to join in with their own responses or improvised lyrics. The singers also used vocal games and call-and-response techniques, such as singing a line and prompting patients to repeat or respond with their own variations. This approach aimed to stimulate interaction and creativity among the participants. The singers adapted their singing and activities to the needs of the participants involved. This meant that sometimes, they would simply talk to someone for a while, if they felt the participant wanted to talk. On other occasions, especially when participants with severe dementia were not responsive, they would simply sit next to someone and sing for them, without expecting the participant to join in. When participants initiated physical contact (e.g., holding hands, dancing together), this was also included in the activity. When available, the singers used various attributes in the room to assist their music making (e.g., drumming on the table, but also playing on a guitar owned by one of the participants). Hence, depending on the participants’ response and capabilities, the activity had an active or more passive nature. The *Stem&Luister* intervention was thus designed specifically to adapt the singing to the needs, preferences, and abilities of the participants. In *Stem&Luister*, participants are targeted individually, and the intervention is designed to be applicable within the range of capabilities of typical residents in a nursing home facility, with various stages of dementia. Note that although every *Stem&Luister* session is fully improvised, *Stem&Luister* follows a clear methodology in how to engage with the residents in a person-centered manner, and in how to develop the musical sessions as a group of singers, similar to how improvisation techniques can be used in a structured manner in musical therapy.

There were no set rules on which singers interacted with specific participants, as they let this happen naturally and freely. In practice, this often meant the same singer would see the same participants each week, as they familiarized with each other. The four singers would attend to residents across the room (i.e., the four singers approached different participants). All participants who wanted to engage with the singers could do so, and additionally, singers encouraged participants to engage. For participants who did not actively engage with the singers, the activity was passive, in the sense that these participants were still exposed to the joint singing, but not actively engaging with the activity. Hence, all residents were exposed to the intervention for the full 50 min, but active engagement depended on their needs and response.

To be capable of responding to the needs of the participants, singers were trained in how to interact with people with dementia by professional staff of the nursing home. Training included approaching people with dementia, interpreting responses by participants, and identifying and dealing with resistance or negative responses. The singers involved in the intervention for this study additionally had extensive experience in working with people with dementia. Prior to each session, the singers prepared together by engaging in vocal and breathing exercises and group coordination activities to ensure they were ready to engage effectively with the participants.^24^

Conversation. The conversation activity was identical to the singing activity, with the singers interacting with participants freely. However, in the conversation activity, singers were instructed not to use song to connect to participants. Instead, they conversed normally. All other parameters were kept identical to the singing sessions (e.g., the same singers, the same observers, the same warming up time, though warm-up consisted of conversation instead of singing).

### Data analysis

Data analysis was performed in R (version 4.3.2).^49^ Given the low power due to the limited sample size for this pilot study, we refrained from drawing conclusions based on conventional statistics. To get some estimate of the reliability of the results, Bayesian statistics were instead implemented. For missing NPI data, where either severity or caregiver distress was missing but frequency was recorded correctly, given the high correspondence between frequency, severity and caregiver distress, the average from other participants with the same frequency at the same timepoint was computed and used. This occurred for 4 data points in total (3 severity values and 1 caregiver distress value, accounting for 0.2% of the total data). Where an item was scored as “yes” (i.e., indicating the presence of symptoms), but scores were missing for all three items (i.e., frequency, severity, and caregiver distress were missing), the average for the same participant from all other timepoints was used. This occurred for 3 different symptoms, in 3 different participants (i.e., the maximum missing data for these participants was from a single symptom, and was imputed from 3 other time points). Missing data for the QUALID was addressed by computing the mean of the item at all other time points of that participant. This occurred for 1 data point (i.e., 1 value from 1 participant, accounting for 0.2% of the total data).

We used the BayesFactor package^50^ to perform Bayesian T-tests to compare 1) the NPI-NH and QUALID scores before and after the singing and conversation activities, for each activity separately as well as for both activities together (i.e., by taking the average of the two activities for each participant) to index the effect of the interventions, 2) the NPI-NH and QUALID difference scores from before to after the intervention between singing and conversation activities, to index differences in the effects of the two activities, and 3) the MPES and affect scores between the singing and conversation sessions, to index differences in engagement and affect during the activities. We regarded as null hypotheses that there would be no difference between outcomes before and after the activities, and no differences between the effects of singing and conversation. A Bayes factor of 1–3 is regarded as anecdotal or inconclusive evidence for the alternative hypothesis (i.e., a difference between conditions or time points), a Bayes factor of 3–10 as moderate evidence, and a Bayes factor of >10 as strong evidence.^51^ Similarly, a Bayes factor of 0.3–1 is considered anecdotal or inconclusive evidence for the null hypothesis, a Bayes factor of 0.1–0.3 moderate evidence, and a Bayes factor of <0.1 strong evidence for the null hypothesis.^51^ We did not compute Bayes factors for the items anger, anxiety, inappropriate behavior, and leaving of the room, as these items contained only a few datapoints, because these behaviors were hardly observed. In addition to Bayes factors, we provide standardized effect sizes (Cohen's d), computed using the R *effectsize* package,^52^ and descriptive statistics to aid future studies.

To assess engagement related to severity of the dementia, we additionally computed correlations between the engagement and affect scores over all sessions and the dementia severity score, as indexed by the BANS-S. Measures for behavior that was observed frequently during the sessions (i.e., constructive and passive engagement, and pleasure) were correlated with BANS-S scores using Pearson's r. We report uncorrected p-values for these correlations. Finally, we conducted a sensitivity analysis removing the participant with acquired brain injury, evaluating whether outcomes changed qualitatively. All materials, data and analysis scripts can be found here: https://osf.io/avx9u/overview?view_only=515042c27a7d4373ba96d42e34399d60.

## Results

### Assessment of activities by interventionists

The mean results of the ratings given by the singers for each session are displayed in Table 1. Overall, no clear difference is visible in the scoring between the singing and conversation sessions, except for the amount of singing in the sessions.

**Table 1.** Singer's experience during the sessions.

	Song		Connect		Response		Active		Satisfied	
	M	SD	M	SD	M	SD	M	SD	M	SD
Singing	3.20	0.82	3.55	0.75	3.88	0.56	3.30	0.61	3.62	0.87
Conversation	1.02	0.16	3.62	0.81	3.75	0.54	3.20	0.72	3.58	0.75

Song = How much did you sing?; Connect = How much did you feel you connected with the participants?; Response = How positively did the participants respond?; Active = How actively engaged were the participants?; Satisfied = How satisfied were you with the session?; M = Mean; SD = Standard Deviation. Answer options ranged from 1 till 5.

### Effects of music and conversation activities on behavioral symptoms and professional caregiver burden (NPI-nh)

*Pre-post comparisons.* The results of the NPI-NH are shown in Figure 2 and Table 2. For the conversation activity, there was anecdotal evidence for the null hypothesis (e.g., no difference between NPI scores before and after the activity) for both behavioral symptoms (BF_10_ = 0.318) and professional caregiver burden (BF_10_ = 0.726). Similarly, for the singing activity, we found anecdotal evidence to support the null hypothesis for behavioral symptoms (e.g., no difference between the NPI total scores before and after the activity; BF_10_ = 0.646). For professional caregiver distress, we found anecdotal evidence for the alternative hypothesis (e.g., lower scores – lower burden – after than before the singing activity; BF_10_ = 1.862). The overall difference between NPI scores before and after the activity (i.e., collapsed over singing and conversation sessions, top of Figure 2) was numerically consistent with an improvement (i.e., lower scores after the intervention), but Bayes factors indicated anecdotal to moderate evidence for the null hypothesis (e.g., no difference from baseline to intervention; BF_10_ = 0.313 for participant NPI total score, and BF_10_ = 0.296 for NPI professional caregiver distress).

**Figure 2.**
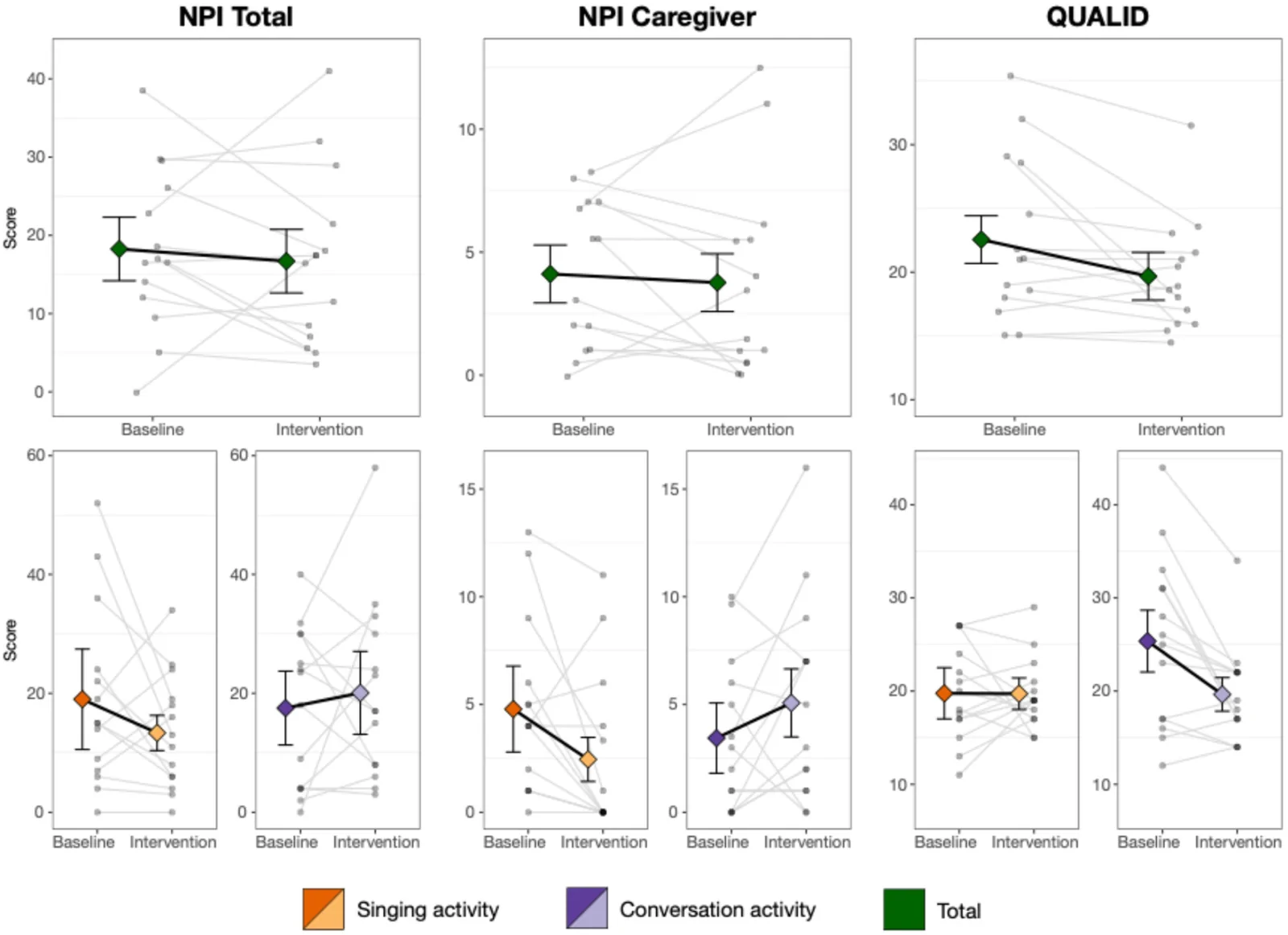
Results for behavioral symptoms, professional caregiver burden (NPI-NH), and participant wellbeing (QUALID). The results of the NPI Total score (participant), NPI Caregiver Distress score (professional caregiver), and QUALID score are displayed, both for all sessions together (top) and separate for singing and conversation (bottom). Higher NPI-NH and higher QUALID scores are unfavorable. Error bars display the 95% confidence interval, adjusted for the within-subject nature of the study using the *SummarySEWithin* function from the Rmisc package.^53^.

**Table 2.** Means, standard deviations, difference scores, and effect sizes for behavioral symptoms, professional caregiver burden (NPI-nh), and participant wellbeing (QUALID).

Measure	Condition	Pre		Post		Diff		Effect size	
		M	SD	M	SD	M	SD	*d*z	95% CI
NPI Total	Combined	18.26	10.38	16.71	11.18	−1.55	9.95	−0.16	[-0.68 0.37]
	Singing	19.00	15.30	13.34	9.79	-5.66	14.50	-0.39	[-0.93 0.16]
	Conversation	17.52	13.37	20.07	15.28	2.55	15.56	0.16	[-0.37 0.69]
NPI Caregiver Distress	Combined	4.11	3.03	3.76	4.03	-0.35	2.88	-0.12	[-0.65 0.41]
	Singing	4.79	4.06	2.45	3.74	-2.33	3.83	-0.61	[-1.17 -0.03]
	Conversation	3.44	3.56	5.07	4.71	1.63	3.90	0.42	[-0.14 0.96]
QUALID	Combined	22.56	6.43	19.68	4.42	-2.88	4.58	-0.63	[-1.19 -0.04]
	Singing	19.76	5.19	19.71	3.83	-0.05	5.11	-0.01	[-0.53 0.51]
	Conversation	25.36	9.33	19.64	5.26	-5.71	6.59	-0.87	[-1.47 -0.24]

Higher scores for NPI and QUALID are unfavorable. Combined scores were obtained by averaging over singing and conversation activities for each participant, before computing a grand average. NPI Total scores represent behavior as observed in participants. NPI Caregiver Distress scores represent perceived burden on professional caregivers. Pre: Baseline score; Post: Score after intervention; Diff: Difference score (post-pre); M: mean; SD: standard deviation; *d*z: Standardized effect size Cohen's d (adjusted for within-subject testing); CI: confidence interval for *d*z (i.e., lower and upper boundary of the 95% confidence interval).

*Music-conversation comparisons.* Regarding the comparison between the effects of the music and conversation activities on behavioral symptoms (the total participant NPI score), we found anecdotal evidence for the null hypothesis (e.g., no difference between conditions in their effect on baseline to intervention difference scores, see Figure 2, BF_10_ = 0.581). For professional caregiver burden, we found moderate evidence for the alternative hypothesis: professional caregiver burden, as measured by the NPI, improved more after singing than after talking (BF_10_ = 4.527).

### Effects of music and conversation activities on wellbeing (QUALID)

*Pre-post comparisons.* The results of the QUALID are shown in Figure 2 and Table 2. For the conversation activity, we found moderate evidence for the alternative hypothesis: scores were lower after the conversation activity than at baseline, consistent with an improvement in quality of life (BF_10_ = 8.192). For singing however, we found moderate evidence for the null hypothesis (e.g., no effect of singing on wellbeing; BF_10_ = 0.27).

*Music-conversation comparisons.* The improvement in QUALID scores was larger after the conversation activity compared to the singing activity (comparison of the difference scores; BF_10_ = 4.448, indicating moderate evidence for the alternative hypothesis). For all data combined, the evidence for a difference between scores at baseline and after the activity (e.g., the alternative hypothesis) was anecdotal (BF_10_ = 2.074).

### Engagement and affect in response to music and conversation activities

MPES and affect scores are shown in Figure 3. Bayes factors were computed for all MPES items comparing the talking with the singing activities. We found anecdotal evidence for the null hypothesis for constructive engagement (BF_10_ = 0.576), and non-engagement (BF_10_ = 0.342), and moderate evidence for the null hypothesis for passive engagement (BF_10_ = 0.296), pleasure (BF_10_ = 0.274) and sadness (BF_10_ = 0.286), suggesting no difference between the scores on these items for the different activities. For other engagement, we found anecdotal evidence for the alternative hypothesis (BF_10_ = 1.338), with more other engagement during singing than conversation.

**Figure 3.**
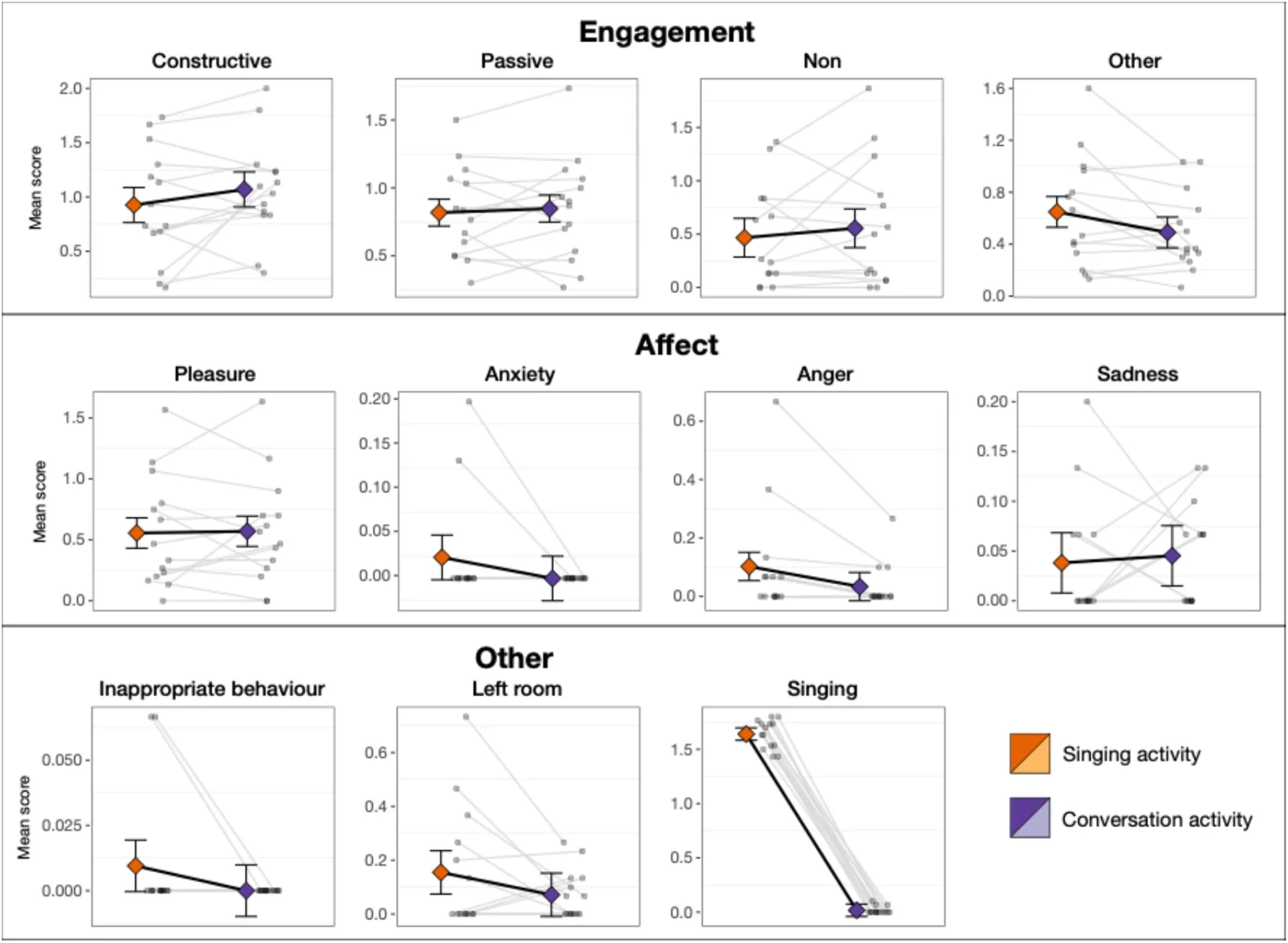
Results for engagement and affect (MPES) and their relationship with dementia severity. The results of each individual element of the MPES are displayed. Elements were scored with a range between 0 and 2. Engagement types are scored in a mutually exclusive way. Error bars display the 95% confidence interval, adjusted for the within-subject nature of the study using the *SummarySEWithin* function from the Rmisc package.^53^

### Relationship between engagement and dementia severity

Figure 4 shows the relationship between severity of the dementia (i.e.,., BANS-S score) and constructive engagement, passive engagement, and pleasure, as indexed by the MPES scores. As is apparent from the figure, there is a negative relationship between dementia severity and constructive engagement (*r* = -0.71, *p* < 0.005) and between dementia severity and pleasure (*r* = -0.71, *p* < 0.005). There is a positive relationship between dementia severity and passive engagement, albeit not significant (*r* = 0.48, *p* = 0.08). The outcomes of any of the analyses did not change qualitatively when removing the participant with acquired brain injury from the analyses.

**Figure 4.**
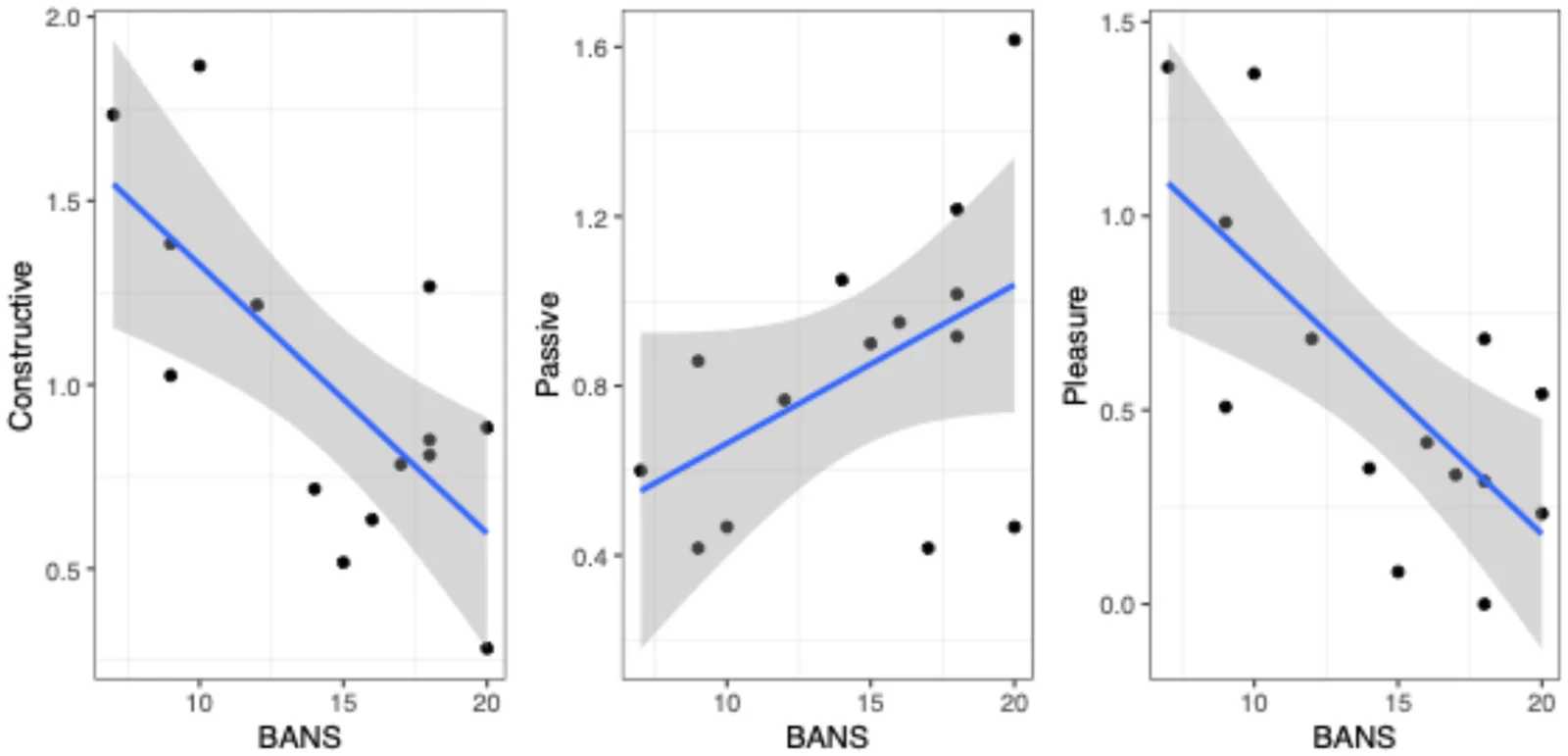
Relationship between constructive and passive engagement and pleasure with dementia severity.

## Discussion

The purpose of our pilot study was to examine the feasibility of our study design for assessing the effects of a singing activity on behavioral symptoms, engagement, quality of life, and caregiver burden in individuals with dementia. To isolate the specific effects of music, we compared a singing activity with a conversational activity, which was identical to the singing in all aspects, except for the presence of music. The sample size of the pilot experiment does not allow us to use conventional statistics to draw conclusions—instead, we opted for computing Bayes factors. To facilitate future studies, we provided effect sizes for all effects of interest. Bayes factors showed only anecdotal evidence for most comparisons, which indicates that the null and alternative hypotheses are almost equally likely (each hypothesis being between 1–3 times more likely than the other hypothesis), and moderate evidence (one hypothesis 3–10 times more likely than the other) for some comparisons. Evidence for the null hypothesis (e.g., no difference between baseline and after the intervention) was found for behavioral symptoms for both singing and conversation activities, as well as for caregiver burden for conversation, and wellbeing for singing. Moreover, evidence for the null hypothesis for singing-conversation comparisons was found for behavioral symptoms, affect during the activities, and constructive, passive, and non-engagement. Hence, the majority of measures showed neither effects of the intervention, nor a difference between the effects of singing and conversation.

We did find moderate evidence for an effect of the singing activity on professional caregiver distress (as assessed with the NPI), and of the conversation activity on quality of life (as assessed using the QUALID). Both effects were in the expected direction, with less professional caregiver distress and better quality of life after the interventions. This highlights the general potential of both interventions for people with dementia. However, first, the baseline scores for the QUALID appeared different between the singing and conversation activities (Figure 2). Hence, the improvement observed in the conversation activity may well have been due to regression to the mean, rather than an effect of the intervention. Second, the current pilot data do not yield strong evidence in favor of conversational or singing activities for improving behavioral symptoms and quality of life in individuals with dementia. Moreover, we found moderate evidence for similar levels of engagement and induced emotions across the two activities, as evidenced by equal scores on the MPES and affect ratings. Together, this suggests that the activities may have been equally successful in eliciting positive effects in people with dementia. These observations from the current pilot study of course need to be confirmed in a higher-powered study.

Overall, this pilot study demonstrated the feasibility of using a randomized crossover design with direct observations by researchers to compare the effects of different types of interactive activities in a dementia care setting. Anecdotally, while the singers initially indicated hesitation at having to lead their activity without actually singing, the results of the questionnaire indicated that they in fact fared equally well in both activities, with no differences in their overall satisfaction, nor in how much they felt their activities were effective at engaging the participants. Also, we observed that they indeed sang during the singing activity but not during the conversational activity. Given that the intervention was designed to be led by trained volunteers, this makes the current setup a feasible one for a larger-scale study with the aim of isolating the specific contributions of music to the effects of musical activities in people with dementia.

In the current pilot study, we found no clear advantage for musical activities over conversational activities for people with dementia. While this finding needs to be confirmed in large-scale studies, it raises questions about the specific mechanisms through which different types of activities influence well-being. While (group) singing activities are generally thought to enhance mood and social connection,^54^ it appears that, in our study, singing did not have a larger effect on outcome measures than conversation, possibly due to the direct and personal nature of verbal interaction. According to Kitwood's (1997) theory of person-centered care, individuals with dementia have psychosocial needs that include comfort, attachment, inclusion, identity, and occupation.^55^ Verbal interaction, especially in a conversational format, may help address these needs by encouraging a sense of connection and validation, and as such, may not be inferior to musical interaction. The putative positive effects of music interventions may as such lie more in the connections between people that music helps foster than in the music itself, or, as recently suggested, in the ability of music to meet several emotional, social, and cognitive needs of people with dementia,^56^ similar to other psychosocial interventions. Whether trained musicians, such as the singers administering the intervention in the current study, may have an advantage in achieving such connectedness through their extensive experience with music in is an interesting topic for future research.

Reschke-Hernández et al. (2023) found a difference between a conversation and a singing activity.^13^ Of note, in their study specific topics were used as discussion starters whereas in our study, the conversational activity was not limited in the scope of topics. Allowing conversation to flow freely around the topics of interest to the participants may have advantaged the conversation activity in the current study. Conversely, while improvised singing may have the benefit of allowing tailoring to the individual participant,^24^ the amount of well-known songs in our intervention was limited, since we used an existing intervention that focuses on improvisation.^24^ However, well-known songs are a powerful way of eliciting autobiographical memory in dementia,^57–59^ and can elicit strong positive emotional responses in people with dementia.^60,61^ Hence, compared with the previous work,^13^ the music activity may have been disadvantaged. The specific characteristics of the interventions may thus explain potential differences in effects. Determining the exact (personalized) ingredients of music interventions that elicit the most positive outcome in people with dementia remains an important topic for future studies.

Given the variable outcomes of previous research looking at the effects of music on neuropsychiatric symptoms and quality of life,^10,62^ as well as the lack of observed difference in the current study, additional research is needed to confirm the specific effects of musical activities on quality of life in people with dementia and to explore the mechanisms underlying these effects. Recently, a large controlled clinical trial aimed towards understanding music interventions in dementia by comparing music therapy with a singing activity and these combined with usual care.^7^ However, no active control group was included, hence the specific effects of music could not be assessed.

Importantly, from our data it becomes apparent that a higher severity of dementia was associated with lower levels of active engagement with the activities, as well as lower levels of overt expressions of pleasure, which is in line with the study of Reschke-Hernández et al. (2023).^13^ Individuals with advanced dementia may not engage actively during activities anymore, often defaulting to more passive states.^45^ This observation has several implications for future research. First, these results underscore the importance of tailoring engagement strategies to the cognitive abilities of participants, as individuals with higher dementia severity may benefit more from passive forms of engagement or multisensory activities that do not demand active participation, for which music interventions lend themselves well.^63^ Indeed, the current intervention was flexibly adapted to have a more active or passive nature, depending on the needs and abilities of the participants. Second, these results may question our assessment of the success of the interventions. Given that we assessed engagement as one of the primary outcome variables, the success of the interventions may not have been visible in participants with more severe dementia, who made up almost half of our sample. Anecdotally, in our study many of the interventionists mentioned special moments of connectedness with the participants, especially while they were singing, and especially in participants with more severe dementia, which they felt were not picked up by our observations (e.g., “There was a very subtle tilting of the head towards the music and some responses in shoulder and fingers.” and “It was so heartwarming”). While this may be due to expectancy or experimenter bias, given that the singers were trained for a singing intervention, not for a conversational intervention, it is also possible that our measurements, while geared towards severe dementia,^64^ were not suitable to pick up the subtle effects of music.^65^ Future studies may consider alternative instruments, such as Dementia Care Mapping,^66^ or qualitative methods.

Interestingly, the recent large-scale study of Baker et al. (2022) found a positive effect of an active music intervention (recreational choir singing) on depressive symptoms in people with dementia, with the largest effects for people with more severe dementia.^7^ This raises questions on what mechanism allowed people with more severe dementia, who in our study showed minimal engagement, to benefit from the activity. One possibility is that it is the social nature of the activity that is crucial.^58^ Future research may examine whether passive engagement in a social musical activity, as is more frequently observed for people with severe forms of dementia, may still provide behavioral benefits, despite overt synchronization, which is often seen as driving the social^67^ and pleasurable^68^ effects of music, being absent in passively engaging individuals.

To conclude, this pilot study illustrates the potential of using a crossover design with a conversation activity as an active control condition to examine the specific effects of music on caregiver burden, behavioral symptoms and quality of life in people with dementia. Moreover, our study, despite its low power, offers some valuable insights for future work. First, our study stresses the necessity to understand the specific effects of music. Second, we highlight how dementia severity may affect the relationship between active engagement and positive effects of musical activities, which may be an interesting topic for future research, especially in the light of exploring different ways of quantifying positive effects of music in people with severe dementia. Finally, our study highlights the need for understanding the exact nature of both singing and conversational activities that leads to the largest benefits, which may come from intervention details such as the use of familiar song, and personal preferences for conversation topics. Taken together, such efforts can further optimize the use of music for well-being in dementia.

## Data Availability

All materials, data and analysis scripts can be found here: https://osf.io/avx9u/overview?view_only=515042c27a7d4373ba96d42e34399d60.

## References

[1] Kirova A-M Bays RB Lagalwar S . Working memory and executive function decline across normal aging, mild cognitive impairment, and Alzheimer’s disease. Biomed Res Int 2015; 2015: 748212. 10.1155/2015/748212 PMC4624908 26550575

[2] Livingston G Huntley J Sommerlad A , et al. Dementia prevention, intervention, and care: 2020 report of the lancet commission. Lancet 2020; 396: 413–446. 10.1016/S0140-6736(20)30367-6 PMC7392084 32738937

[3] Antipas H Tamplin J Vieira Sousa T , et al. Interventions for mitigating occupational stress for professional dementia caregivers in residential aged care: a systematic review with meta-analysis. Dementia 2024; 23: 292–311. 10.1177/14713012231220963 PMC10807264 38069510

[4] Sikkes SAM Tang Y Jutten RJ , et al. Toward a theory-based specification of non-pharmacological treatments in aging and dementia: focused reviews and methodological recommendations. Alzheimers Dement 2021; 17: 255–270. 10.1002/alz.12188 PMC7970750 33215876

[5] Särkämö T Sihvonen AJ . Golden oldies and silver brains: deficits, preservation, learning, and rehabilitation effects of music in ageing-related neurological disorders. Cortex 2018; 109: 104–123. 10.1016/j.cortex.2018.08.034 30312779

[6] Zatorrea RJ Salimpoor VN . From perception to pleasure: music and its neural substrates. Proc Natl Acad Sci U S A 2013; 110: 10430–10437. 10.1073/pnas.1301228110 PMC3690607 23754373

[7] Baker FA Lee Y-EC Sousa TV , et al. Clinical effectiveness of music interventions for dementia and depression in elderly care (MIDDEL): Australian cohort of an international pragmatic cluster-randomised controlled trial. Lancet Healthy Longev 2022; 3: e153–e165. 10.1016/S2666-7568(22)00027-7 36098290

[8] Cho HK. The effects of music therapy-singing group on quality of life and affect of persons with dementia: a randomized controlled trial. Front Med (Lausanne) 2018; 5: 279. 10.3389/fmed.2018.00279 PMC6232901 30460234

[9] Gold C Eickholt J Assmus J , et al. Music interventions for dementia and depression in ELderly care (MIDDEL): protocol and statistical analysis plan for a multinational cluster-randomised trial. BMJ Open 2019; 9: e023436. 10.1136/bmjopen-2018-023436 PMC6475205 30928926

[10] van der Steen JT van der Wouden JC Methley AM , et al. Music-based therapeutic interventions for people with dementia. Cochrane Database Syst Rev 2025; 2025: CD003477. 10.1002/14651858.CD003477.pub5 PMC11884930 40049590

[11] Wetzels RB Zuidema SU de Jonghe JFM , et al. Determinants of quality of life in nursing home residents with dementia. Dement Geriatr Cogn Disord 2010; 29: 189–197. 10.1159/000280437 20215750

[12] Santini S Merizzi A Azevedo MJ , et al. Effects of a person-centered music-based intervention in the rehabilitation of older adults with mild to moderate dementia. J Alzheimers Dis Rep 2025; 9: 25424823251367292. 10.1177/25424823251367291 PMC12437183 40963978

[13] Reschke-Hernández AE Gfeller K Oleson J , et al. Music therapy increases social and emotional well-being in persons with dementia: a randomized clinical crossover trial comparing singing to verbal discussion. J Music Ther 2023; 60: 314–342. 10.1093/jmt/thad015 PMC10560009 37220880

[14] Maseda A Cibeira N Lorenzo-López L , et al. Multisensory stimulation and individualized music sessions on older adults with severe dementia: effects on mood, behavior, and biomedical parameters. J Alzheimers Dis 2018; 63: 1415–1425. 10.3233/JAD-180109 29843244

[15] Narme P Clément S Ehrlé N , et al. Efficacy of musical interventions in dementia: evidence from a randomized controlled trial. J Alzheimers Dis 2014; 38: 359–369. 10.3233/JAD-130893 23969994

[16] Abeywickrama N Miraval MNE Subramaniam H , et al. Efficacy of music-based intervention for people living with dementia in an inpatient setting: a pilot study. J Alzheimers Dis 2025; 103: 905–919. 10.1177/13872877241307311 39801125

[17] Coxey JP Kameg B Novosel LM , et al. Music and nursing home residents with dementia: a literature review. J Nurse Pract 2021; 17: 808–814.

[18] van der Steen JT Smaling HJ van der Wouden JC , et al. Music-based therapeutic interventions for people with dementia. Cochrane Database Syst Rev 2018; 2018: CD003477. 10.1002/14651858.CD003477.pub4 PMC6513122 30033623

[19] Samson S Clément S Narme P , et al. Efficacy of musical interventions in dementia: methodological requirements of nonpharmacological trials. Ann N Y Acad Sci 2015; 1337: 249–255. 10.1111/nyas.12621 25773641

[20] Clément S Tonini A Khatir F , et al. Short and longer term effects of musical intervention in severe Alzheimer’s disease. Music Percept 2012; 29: 533–541.

[21] Bruscia KE. An introduction to music psychotherapy. In: The Dynamics of Music Psychotherapy. Barcelona Publishers (NH), 1998, pp.1–15.

[22] Dileo C Bradt J . Medical Music Therapy: A Meta-analysis & Agenda for Future Research. Jeffrey Books, 2005.

[23] Sousa L Neves MJ Moura B , et al. Music-based interventions for people living with dementia, targeting behavioral and psychological symptoms: a scoping review. Int J Geriatr Psychiatry 2021; 36: 1664–1690. 10.1002/gps.5568 34097789

[24] Gysels M Tonelli C Johannsen T . Making dementia matter through sound. Voices 2024; 24: 1–25.

[25] Dahms R Eicher C Haesner M , et al. Influence of music therapy and music-based interventions on dementia: a pilot study. J Music Ther 2021; 58: e12–e36. 10.1093/jmt/thab005 33948656

[26] Jones C Sung B Moyle W . Engagement of a person with dementia scale: establishing content validity and psychometric properties. J Adv Nurs 2018; 74: 2227–2240. 10.1111/jan.13717 29772602

[27] Su E Kenway L . The impact of music therapy on cognitive abilities in patients with Alzheimer’s disease and related dementias: a systematic review. Br J Music Ther 2026; 40: 38–52.

[28] Bian X Wang Y Zhao X , et al. Does music therapy affect the global cognitive function of patients with dementia? A meta-analysis. Neurorehabilitation 2021; 48: 553–562. 10.3233/NRE-210018 33967069

[29] Bugos JA Lu L Chen L , et al. The effects of active music interventions on cognitive function and neuropsychiatric symptoms in patients with dementia: a systematic review and meta-analysis. Music Sci 2024; 28: 112–130.

[30] Särkämö T Tervaniemi M Laitinen S , et al. Cognitive, emotional, and social benefits of regular musical activities in early dementia: randomized controlled study. Gerontologist 2014; 54: 634–650. 10.1093/geront/gnt100 24009169

[31] Kaufer DI Cummings JL Christine D , et al. Assessing the impact of neuropsychiatric symptoms in Alzheimer’s disease: the neuropsychiatric inventory caregiver distress scale. J Am Geriatr Soc 1998; 46: 210–215. 10.1111/j.1532-5415.1998.tb02542.x 9475452

[32] Schalkwijk D Verlare LR Muller MT , et al. Measuring quality of life in nursing home residents with severe dementia: psychometric properties of the QUALID scale. Tijdschr Gerontol Geriatr 2009; 40: 184–192. 10.1007/BF03079587 21140954

[33] Weiner MF Martin-Cook K Svetlik DA , et al. The quality of life in late-stage dementia (QUALID) scale. J Am Med Dir Assoc 2000; 1: 114–116. 12818023

[34] de Jonghe JFM Kat MG Kalisvaart CJ , et al. Neuropsychiatric inventory questionnaire (NPI-Q): a validity study of the Dutch form. Tijdschr Gerontol Geriatr 2003; 34: 74–77. 12741091

[35] Kat MG De Jonghe JFM Aalten P , et al. Neuropsychiatrische symptomen bij dementie: psychometrische aspecten van de nederlandse neuropsychiatric inventory (NPI). Tijdschr Gerontol Geriatr 2002; 33: 150–155. 12378786

[36] Kaufer DI Cummings JL Ketchel P , et al. Validation of the NPI-Q, a brief clinical form of the neuropsychiatric inventory. J Neuropsychiatry Clin Neurosci 2000; 12: 233–239. 10.1176/jnp.12.2.233 11001602

[37] Wood S Cummings JL Hsu MA , et al. The use of the neuropsychiatric inventory in nursing home residents: characterization and measurement. Am J Geriatr Psychiatry 2000; 8: 75–83. 10.1097/00019442-200002000-00010 10648298

[38] Volicer L Hurley AC Lathi DC , et al. Measurement of severity in advanced Alzheimer’s disease. J Gerontol 1994; 49: M223–M226. 10.1093/geronj/49.5.m223 8056941

[39] Appollonio I Gori C Riva G , et al. Assessing early to late stage dementia: the TSI and BANS-S scales in the nursing-home. Int J Geriatr Psychiatry 2005; 20: 1138–1145. 10.1002/gps.1406 16315161

[40] Bellelli G Frisoni GB Bianchetti A , et al. The bedford Alzheimer nursing severity scale for the severely demented: validation study. Alzheimer Dis Assoc Disord 1997; 11: 71–77. 10.1097/00002093-199706000-00003 9194953

[41] van der Steen JT Volicer L Gerritsen DL , et al. Defining severe dementia with the Minimum data set. Int J Geriatr Psychiatry 2006; 21: 1099–1106. 10.1002/gps.1618 16955417

[42] Cummings JL Mega M Gray K , et al. The neuropsychiatric inventory: comprehensive assessment of psychopathology in dementia. Neurology 1994; 44: 2308–2314. 10.1212/wnl.44.12.2308 7991117

[43] Bužgová R Kozáková R Sikorová L , et al. Measuring quality of life of cognitively impaired elderly inpatients in palliative care: psychometric properties of the QUALID and CILQ scales. Aging Ment Health 2017; 21: 1287–1293. 10.1080/13607863.2016.1220919 27565013

[44] Resnick B Galik E Kolanowski A , et al. Reliability and validity testing of the quality of life in late-stage dementia scale. Am J Alzheimers Dis Other Demen 2018; 33: 277–283. 10.1177/1533317518765133 PMC6391977 29575907

[45] Camp CJ Skrajner MJ . Resident-assisted montessori programming (RAMP): training persons with dementia to serve as group activity leaders. Gerontologist 2004; 44: 426–431. 10.1093/geront/44.3.426 15197297

[46] Judge KS Camp CJ Orsulic-Jeras S . Use of montessori-based activities for clients with dementia in adult day care: effects on engagement. Am J Alzheimers Dis Other Demen 2000; 15: 42–46.

[47] Skrajner MJ Camp CJ . Resident-Assisted montessori programming (RAMP^™^): use of a small group Reading activity run by persons with dementia in adult day health care and long-term care settings. Am J Alzheimers Dis Other Demen 2007; 22: 27–36. 10.1177/1533317506297895 PMC10697202 17533999

[48] Lawton MP Van Haitsma K Klapper J . Observed affect in nursing home residents with Alzheimer’s disease. J Gerontol B Psychol Sci Soc Sci 1996; 51: P3. 10.1093/geronb/51b.1.p3 8548515

[49] R Core Team. R: A Language and Environment for Statistical Computing. https://www.R-project.org/ (2023, accessed 17 December 2024 ) .

[50] Morey R Rouder J. Computation of Bayes Factors for Common Designs. https://CRAN.R-project.org/package=BayesFactor (2024).

[51] van Doorn J van den Bergh D Böhm U , et al. The JASP guidelines for conducting and reporting a Bayesian analysis. Psychon Bull Rev 2021; 28: 813–826. 10.3758/s13423-020-01798-5 PMC8219590 33037582

[52] Ben-Shachar M Lüdecke D Makowski D . Effectsize: estimation of effect size indices and standardized parameters. J Open Source Softw 2020; 5: 2815.

[53] Hope RM. Rmisc: Ryan Miscellaneous. R package version 1.5.1. https://CRAN.R-project.org/package=Rmisc (2022).

[54] Good A Russo FA . Changes in mood, oxytocin, and cortisol following group and individual singing: a pilot study. Psychol Music 2022; 50: 1340–1347.

[55] Kitwood T. Dementia reconsidered: The Person Comes First. Open University Press, 1997.

[56] Thompson N Odell-Miller H Underwood BR , et al. How and why music therapy reduces distress and improves well-being in advanced dementia care: a realist review. Nature Mental Health 2024; 2: 1532–1542.

[57] Baird A Brancatisano O Gelding R , et al. Music evoked autobiographical memories in people with behavioural variant frontotemporal dementia. Memory 2020; 28: 323–336. 10.1080/09658211.2020.1713379 31959062

[58] Christensen J Timmers R MacRitchie J . Reassessing the goals of musical activities for people living with dementia: supporting joint agency, selfhood and couplehood with an embodied and relational approach. J Aging Stud 2025; 72: 101289. 10.1016/j.jaging.2024.101289 39993879

[59] Kaiser AP Berntsen D . The cognitive characteristics of music-evoked autobiographical memories: evidence from a systematic review of clinical investigations. Wiley Interdiscip Rev Cogn Sci 2023; 14: e1627. 10.1002/wcs.1627 36223919

[60] Garrido S Dunne L Stevens C , et al. Music playlists for people with dementia: qualitative evaluation of a guide for caregivers. Sci Prog 2021; 104: 368504211014353. 10.1177/00368504211014353 PMC10455001 33929900

[61] Ekra EMR Dale B . Systematic use of song and music in dementia care: health care providers’ experiences. J Multidiscip Healthc 2020; 13: 143–151. 10.2147/JMDH.S231440 PMC7023855 32103974

[62] Zhang Y Cai J An L , et al. Does music therapy enhance behavioral and cognitive function in elderly dementia patients? A systematic review and meta-analysis. Ageing Res Rev 2017; 35: 1–11. 10.1016/j.arr.2016.12.003 28025173

[63] Sittler MC Worschech F Wilz G , et al. Psychobiological mechanisms underlying the health-beneficial effects of music in people living with dementia: a systematic review of the literature. Physiol Behav 2021; 233: 113338. 10.1016/j.physbeh.2021.113338 33497696

[64] Volicer L van der Steen JT . Outcome measures for dementia in the advanced stage and at the end of life. Adv Geriatr 2014; 1: 346485.

[65] Dowson B McDermott O Schneider J . What indicators have been used to evaluate the impact of music on the health and wellbeing of people with dementia? A review using meta-narrative methods. Maturitas 2019; 127: 26–34. 10.1016/j.maturitas.2019.06.001 31351517

[66] Fossey J Lee L Ballard C . Dementia care mapping as a research tool for measuring quality of life in care settings: psychometric properties. Int J Geriatr Psychiatry 2002; 17: 1064–1070. 10.1002/gps.708 12404656

[67] Savage PE Loui P Tarr B , et al. Music as a coevolved system for social bonding. Behav Brain Sci 2021; 44: e59. 10.1017/S0140525X20000333 32814608

[68] Fiveash A Ferreri L Bouwer FL , et al. Can rhythm-mediated reward boost learning, memory, and social connection? Perspectives for future research. Neurosci Biobehav Rev 2023; 149: 105153. 10.1016/j.neubiorev.2023.105153 37019245

